# Balance Rehabilitation in Patients with Traumatic Brain Injury: A Scoping Review

**DOI:** 10.3390/medicina62081453

**Published:** 2026-07-27

**Authors:** Emanuela Ricci, Anna Corru, Gianluca Ciardi, Gianfranco Lamberti

**Affiliations:** 1 Degree Course in Physiotherapy, Department of Medicine and surgery, University of Parma, Piacenza training Centre, Viale Abruzzo 12 B/C, 29017 Fiorenzuola d’Arda, PC, Italy; anna.corru@studenti.unipr.it (A.C.); gianfranco.lamberti@unipr.it (G.L.); 2 Surgical and Rehabilitation Department, Guglielmo da Saliceto Hospital, Azienda Usl of Piacenza, Via Taverna 49, 29121 Piacenza, PC, Italy; 3 Department of Social and Health Professions, Guglielmo da Saliceto Hospital, Azienda Usl of Piacenza, Via Taverna 49, 29121 Piacenza, PC, Italy; 4 University Led Operative Unit of Rehabilitation Medicine and Hospital-Territorial Integration, Department of Rehabilitation, Azienda Usl of Piacenza, Via Caffi 10, 29100 Piacenza, PC, Italy

**Keywords:** traumatic brain injury, physical therapy, balance, rehabilitation, postural stability

## Abstract

*Background and Objectives*: Traumatic brain injury (TBI) is one of the leading causes of long-term disability among adults, with consequences for balance, postural stability, and quality of life. Addressing balance disorders in TBI patients requires a tailored and continuous care approach; the existing literature underlines a general framework for physiotherapy treatments, but no clear profile of technique, dosage and timing of intervention emerges. The present scoping review, therefore, aimed at mapping physiotherapy approaches to balance in TBI population, highlighting their potential effects and role through care pathways. *Materials and Methods*: A scoping review was conducted, according to PRISMA Extension for Scoping Reviews, with the aim of mapping physical therapy approaches to treat balance disorders in adult patients with TBI. A specific search string for each database was formulated through a PCC (Population, Concept, Context) framework. The search, limited to articles in English and published between 2015 and 2025, was conducted across five databases (PubMed, PEDro, Scopus, Embase, and Web of Science), including experimental studies, observational designs, and `case reports. *Results*: From the initial selection of 1052 records, six studies were included: five randomized controlled trials and a case report, published between 2016 and 2022. Results presented high heterogeneity of physiotherapy programs and outcome variations; interventions identified included virtual reality, video game therapy, adapted physical exercise, ballistic training, rapid-resistance elliptical training, and rhythmic auditory stimulation. These studies reported variable improvements in balance and mobility, not even with significant inter-group differences. *Conclusions*: Several techniques showed preliminary evidence in pre/post improvements of balance for TBI patients, including strength exercise, aerobic training and virtual-mediated approaches such as video game therapy and virtual reality. Rhythmic auditory stimulation has unclear effects. Future studies are needed to define the impact of each balance recovery strategy according to the baseline characteristics of the patient.

## 1. Introduction

Traumatic brain injury (TBI), defined as non-degenerative and non-congenital brain damage, is caused by an external physical force [1,2] and is one of the leading causes of long-term disability in people aged between 1 and 45 [3,4]. Global estimates show a rising trend for TBI incidence in recent years, with an average of 259 cases and a prevalence of 448 cases per 100,000 people [5]; falls and road accidents cover the majority of TBI causes [3].

From a clinical point of view, according to the Glasgow Coma Scale (GCS), TBI is clinically classified as mild (GCS 13–15), moderate (GCS 9–12) or severe (GCS 3–8) [6]. These severity levels relate to estimated rates of permanent disability of 10%, 60% and 100% respectively, with an overall mortality rate of between 20% and 30% [7]. Although a recurring set of TBI symptoms are recognized, heterogeneity of injury mechanism, pathophysiology, anatomical location and trauma severity make it more complex to establish a diagnosis, formulate a prognosis and plan treatments [8].

TBI frequently entails motor consequences of varying severity, including deficits in balance and postural control, which may persist in the long term and significantly decrease patients’ functional independence and quality of life [9]. Symptoms such as dizziness, unsteadiness and loss of balance relate to a deficit in sensory integration processes of visual, proprioceptive and/or vestibular information. Balance deficits observed in TBI patients may result from lesions in different cortical areas involved in motor control; in particular, motor cortex damage impairs voluntary control of movements required for dynamic balance; alterations in subcortical and cerebellar structures interfere with the integration of sensory information and posture regulation. Taken together, these impairments reduce the limits of stability, and increase the patient’s vulnerability to loss of balance and falls, with a negative impact on self-efficacy and quality of life [10].

Physiotherapy represents a central tool to effectively manage TBI patients in all phases of care pathway; actually, the literature shows profound limits on physical therapy modalities; however, some major intervention domains have been identified, particularly task-guided approaches, mobility and gait recovery, and computer-based interventions [11]. Within this context, Bridges Guidelines [12], developed in Australia in 2024, provide a general framework to prescribing and promoting physical activity in people with moderate-to-severe head injury across the entire continuum of care. Despite this general guideline, the concept of balance rehabilitation in patients with TBI remains a useful rationale in absence of clear intervention strategies; moreover, the appropriate setting in which conduct balance rehabilitation remains unclear (inpatient intensive treatment or outpatient/home setting, forexample). Starting with this rationale, we conducted a scoping review, to explore and map the available scientific evidence regarding rehabilitation approaches used to treat balance disorders in adult patients with TBI in the chronic phase.

## 2. Materials and Methods

This scoping review aimed to map and summarize current rehabilitation approaches for adult patients with traumatic brain injury, with a focus on physiotherapy interventions aimed at restoring balance. Particularly, the variety of proposed rehabilitation approaches, methods of delivery, treatment components and any reported effect relating to improvements in postural control (in both static and dynamic conditions)/balance and gait were analyzed. A conceptual framework was derived by PRISMA Extension for Scoping Reviews [13], followed as the methodological guide for the entire review conduction.

A search string was formulated following the PCC model, in order to identify potentially includible studies:

Population: Adults (more than 18 years) with diagnosis of TBI and balance disorders.

Concept: Rehabilitation treatments delivered through manual or instrumental techniques.

Context: Intensive inpatient/outpatient setting; home setting.

A literary search was carried out through the following databases: PubMed, PEDro, Scopus (Elsevier), Embase and Web of Science. The search started on 24 June 2025, and last access to all database and final extraction of results was performed on 15 September 2025. Search queries were formulated according to the scheme shown in the Supplementary Material Table S1; snowball citation searching was performed to include further studies from reference by relevant papers. Selection of records was limited to papers published by 2015 to 2025, in English. Quantitative and mixed-methods studies were considered eligible, including RCTS, observational studies, case report/case series; grey literature was excluded from the review process.

Among exclusion criteria, studies regarding children (<18 years) and central nervous system diseases other than TBI (stroke, Parkinson, multiple sclerosis, or amyotrophic lateral sclerosis) were not included; studies regarding concussions were also excluded. No exclusion of physiotherapy approach or study setting was considered. Screening and selection of studies were carried out by two independent reviewers, following identification, screening, eligibility and inclusion stages, in accordance with the PRISMA Statement flowchart [14]; all conflicts were addressed through research team discussion. Following duplicates removal, data were organized using Zotero 8.0 (Digital Scholar, Falls Church, Virginia, USA). An initial screening based on title was carried out, followed by abstract evaluation. Articles deemed suitable at this stage were then retrieved in full-text, to allow for a more in-depth analysis, after which a final review was carried out for included papers. According to JBI recommendations for scoping review conduction [15] and S.A.L.S.A (search, appraisal, synthesis and analysis) framework [16], no methodological evaluation was conducted.

## 3. Results

**Figure 1:**
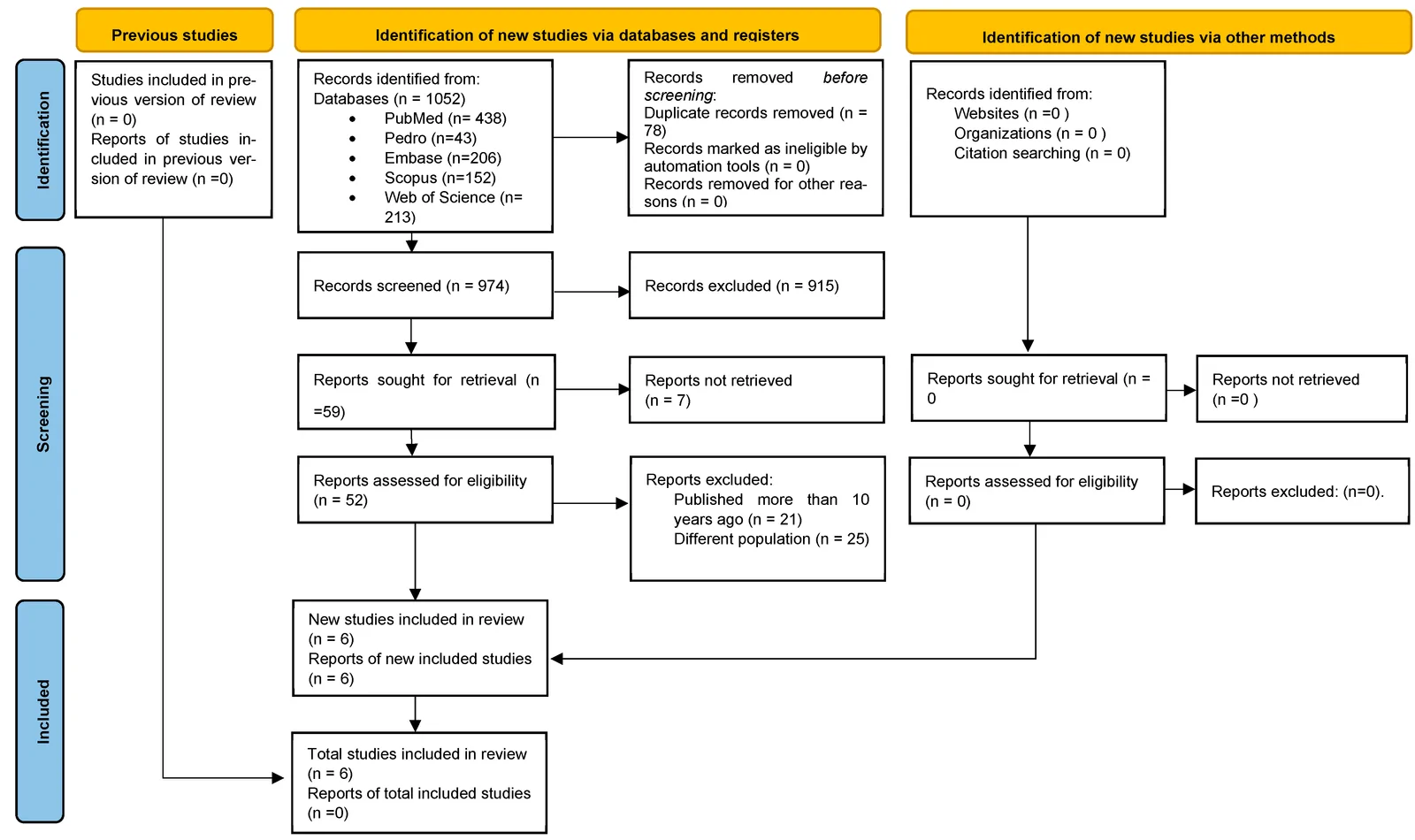
Prisma flowchart of inclusion for extracted results.

The search conducted across five selected databases yielded a total of 1052 records, from which 78 duplicates were removed. From the remaining 974 records, 915 were excluded according to relevance by title and abstract; 59 studies were considered eligible for full text retrieval. Of these, seven were not retrieved, so 52 full-length papers were read. According to eligibility criteria, six studies were included at the final review stage, of which there were five randomized controlled trials and one case report study, published in between 2016 and 2022. Figure 1 represents the Prisma flowchart of inclusion, while Table 1 resumes the main findings from all included studies (data extraction table).

Studies included in the present scoping review showed a common profile of inclusion: TBI diagnosis [17,18,19,20,21,22], ability to independently walk without support [17,18,19,20,21] (in one case ability to walk m unassisted [22]), no recent falls [17], and chronic phase of TBI [17,18,19,20,22]. Only in one case were patients less than 1 year by diagnosis recruited [21]. All studies aimed at testing a physical exercise protocol and its effects on balance, studying overall balance performance [18,19,20,21,22], muscular function [18,19,20,21], attentional abilities [18], gait [22] and field/laboratory tests [17,21,22]. Setting varied among home [17,18,19,20], outpatient services [18,19,21] and community setting [22].

Physiotherapy/exercise protocols widely differed among authors, even in terms of technique and timing of delivering. Three authors tested adapted exercise programs [17,18,21], performed through an elliptic trainer [17], an integrated schedule of resistance, strengthening and stretching training [18], and ballistic resistance training [21]. In particular, Damiano et al. [17] compared a group of chronic TBI patients with healthy volunteers, performing an 8-week exercise program based on an elliptic trainer. Each session involved 30 min of training, aimed at achieving and maintaining a constant, sustained pace (40–80 revolutions per min, corresponding to 80–160 steps/min) for the entire session. Romanov et al. [18], instead, proposed two different programs: all TBI patients followed a standard rehabilitation program (5 days/week for 45 min of endurance exercises, muscle strengthening, and stretching); in addition, a training called ‘Gymnastics for the Brain’ was offered twice a week (a series of simple movements designed to promote activation in both hemispheres). The experimental group also undertook an adapted exercise program twice a week for 90 min (30–40 of Nordic walking using poles with integrated elastic bands, followed by 50–60 of strength and balance exercises). In the third study [21] participants of the experimental group undertook a ballistic resistance program, designed to stimulate muscle strength (jumps with leg extensions and calf raises on a ‘leg sled’, and climbing stairs), and rapid cyclic hip/knee flexion in a standing position. The control group, on the other hand, was offered a traditional program, comprising balance exercises (both static, such as single-leg or tandem standing, and dynamic, such as figure-of-eight walking or heel-to-toe walking), stretching for major muscle groups, conventional strengthening using leg press with high resistance, and slow-execution cardiovascular activity.

Results from these three studies demonstrated a positive direction of change for balance outcome, not even reaching significance: Romanov [18] did not show significant inter-group differences despite balance improvements for all patients, while Damiano [17] demonstrated a significant pre/post reduction in reaction time (*p* = 0.03) and forward excursion (*p* = 0.001) in TBI patients. Williams [21] found a limited (three points) gain in mobility and functional performance at HiMAT (high level mobility assessment) tool for TBI patients, in a general framework of improvement.

An interesting perspective emerged about the use of video game therapy (VGT) and Virtual Reality (VR), from studies of Straudi [19], and Tefertiller [20].

In the first study [19], the experimental group underwent VR training through Xbox 360 Kinect console (Microsoft, Redmond, WA, USA), with focus on dynamic tasks (side steps, weight shifts, jumps, walking in different directions and reaching targets). The control group, instead, underwent a rehabilitation program using a stabilometry platform (Biodex Medical Systems, Inc., Shirley, NY, USA), focusing on postural stability and weight-shifting exercises, performed both with and without visual feedback. Tefertiller et al. [20] confronted a standard home exercise program with a VR one, built through Kinect games. Also, in this case, exercises focused on dynamic activities in standing position, and the tasks’ complexity was determined according to results at functional evaluation (Balance Evaluation Systems Test—BESTest—and the Community Balance and Mobility Scale-CB&M scores). Again here, these studies reported a significant pre/post change in both groups, without inter-group significance, except for the Timed Up and Go (TUG, *p* < 0.01) and Unipedal Balance Test (UBS, *p* < 0.01) reported by Straudi [19].

Finally, the double case report by Sheridan et al. [22] introduced an intervention based on Rhythmic Auditory Stimulation (RAS). The protocol was tailored to each participant’s ability, but all sessions followed a common structure: 30-min sessions, divided into 10 min of preparatory walking exercises, 10 min of specific walking training, 5 min of advanced exercises adapted to the participant’s functional level (e.g., walking on different surfaces, climbing stairs, overcoming obstacles) and 5 min of activities at a therapist’s discretion. Musical recordings, set to predetermined frequencies, were accompanied by metronome beats. Results observed varied: the first participant showed clinically significant improvements in CB&M scores and walking endurance, whilst the second showed only minimal changes, not exceeding the threshold for clinical significance.

## 4. Discussion

The present scoping review aimed at mapping, according to the recent literature, the role and tools of balance rehabilitation in TBI patients, clarifying setting and frequency of intervention. Starting by this scenario, our included studies showed three main chapters of this rehabilitation challenge: resistance and aerobic training, technological devices (VR, VGT and robotic platform) and RAS. Although interventions were different, a common endpoint was safety and a positive pre/post direction of change for interest outcomes. Inter-group differences, instead, showed a heterogeneous and limited profile.

The general theoretical framework for rehabilitation of TBI [12] recommends structuring a continuum of cares; recommended exercise modalities are aerobic, strength training, task-specific training. Rehabilitation programs should include sessions lasting between 15 and 60 min, with a frequency of 3–5 sessions per week for periods ranging from 4 to 12 weeks. Exercise prescription, in addition, should be based on FITT principle (Frequency, Intensity, Time and Type), according to each patient’s abilities, extending them to home setting if adequately supervised.

In line with these principles, some of our results deepened strength and endurance training [18,21], aerobic exercise [17], ballistic exercise [21], not for their priority indication (increasing strength and endurance), but to improve balance; all these strategies seemed to show a positive effect, although not even with statistical inter-group significance. In neurorehabilitation it is well known the principle that interventions focused on muscle contraction and empowerment increase balance and stability [23,24], even through the combination of aerobic and strength strategies; our results confirm this principle even in a TBI population.

Some interesting technology approaches also emerged to improve balance in TBI, particularly innovative treatments based on VR and VGT. Studies included in this review [19,20] provided the same gaming environment to train balance: through the Xbox 360 body tracking system, authors were in fact able to realize an immersive virtual reality to perform dynamic tasks. In addition, the control group in Straudi et al.’s work [19] demonstrated that a stabilometry platform could also effectively improve balance with a lower impact in terms of field test (and so of functional performance).

Overall, both studies confirmed that using VR and VGT correlates with pre/post improved balance performance, in one case also with significance for field tests [19]. These results suggest that VR and VGT may represent useful supplementary interventions for balance recovery in TBI, since qualitative and methodological variability of studies does not, to date, allow them to be considered stand-alone treatments.

Finally, RAS represents an innovative approach that differs markedly from the others in terms of nature and method of application. Despite its primary use in the context of Parkinson’s disease rehabilitation as an external cue [25,26,27], the double case report by Sheridan [22] examined RAS feasibility and impact on mobility in chronic TBI. Despite the safety and good tolerability of RAS intervention, effects were limited to just one of the two patients, so its efficacy remains only purposive and new consistent data should prove it.

The present review had some main limitations to be discussed: firstly, the limited availability of studies and their small sample sizes, which limited generalizability of our results. In addition, there was a lack of correlation between some anamnestic data and TBI patients’ performance: our final population was composed of patients with a history of TBI and the ability to walk independently, regardless of severity of trauma or persistency of consciousness disorders after the injury. Despite the general positive direction of change with different therapeutic tools, heterogeneity of interventions and lack of inter-group significance does not allow us to clarify the true effectiveness of used techniques. Lastly, the different levels of methodological validity of our included studies varied at the lower level (a double case report) of RCTs, thus making ineffective some possible confronts (i.e., auditory training versus VGT).

## 5. Conclusions

This scoping review enabled us to explore the available literature on physiotherapy approaches used to treat balance disorders in adult patients with TBI. Despite small available evidence, some interventions were mapped according to the nature of our work, with a preliminary profile of safety, comprising aerobic training, endurance training, muscle strengthening and virtual reality. Despite a general trend of balance improvement (even in control arms), limited significance was reached for inter-group comparisons, so our results do not define a precise technique or dosage of physiotherapy treatment.

In line with our findings, future studies should better define the impact of each balance recovery strategy according to baseline patient characteristics (i.e., according to the initial GCS score or duration of consciousness loss), thereby creating a tailored approach based on level of impairment. Another powerful aim should be defining balance treatment goals from the subacute phase to the chronic one, to drive neuroplasticity and new motor learning strategies. Finally, a central issue to address will be maintenance of results in the long term; follow-up should clarify outcome maintenance once the patient has been reintegrated into their own family and social environment.

## Figures and Tables

**Table 1:** Data extraction table of included studies; for each study first author and year, design, aim, outcome measure, setting and country, population, intervention and results were presented. Abbreviations: TBI: traumatic brain injury; RCT: randomized controlled trial; EXP: experimental group; CONT: control group; LOS: limits of stability; DT: dual task; min: min; CB&M: Community Balance & Mobility Scale; UBS: Unified Balance Scale; TUG: timed up and go test; IQR: inter-quartile range; VGT: VideoGame Therapy; BESTest: Balance Evaluation Systems Test; ABC: Activities-Specific Balance Confidence Scale; HEP: home exercise program; PART-O test: Participation Assessment with Recombined Tools-Objective; HiMat tool: High Level Mobility Assessment Tool; AQoL-6D: Assessment of Quality of Life; RAS: rhythmic auditory stimulation; RPE: rating of perceived exertion; MCD: minimal clinically important difference.

Author, Year	Design	Aim(s)	Outcome	Setting and Country	Population	Intervention	Results
Damiano et al. 2016 [17]	RCT	Increasing motor and cognitive elaboration speed in walking TBI patients through elliptical trainer with quick resistance.	HiMAT tool, LOS test, DT performance, neurobehavioral questionnaire.	Home setting (USA).	EXP: 12 chronic TBI patients; mean age 31.3 ± 9.4 years. CONT: 12 healthy volunteers; mean age 32.5 ± 9.3 years.	Both groups followed a training program with an elliptical trainer, with the aim to reach and maintain a high stable rhythm during the entire session. Training speed was gradually increased. 30 min sessions were held 5 times/week for 8 weeks.	Patients with TBI had initial balance deficits with limited excursion in LOS test. After the training significant improvements in reaction time (*p* = 0.03) and forward excursion (*p* = 0.001) at LOS test were detected in TBI group. Gain in balance corresponded to reduction of self-reported anxiety and better motor performance.
Romanov et al. 2021 [18]	RCT	Investigating the effects of adapted physical exercise on balance, stability and attentive skills in TBI patients, compared to a standard rehabilitation program.	Balance Berg test, D2 test for attention abilities.	Outpatient specialized center of Maribor (Slovenia).	EXP: 13 men with chronic TBI (more than 2 years by diagnosis) CONT: 12 men with chronic TBI (more than 2 years by diagnosis) Mean age for the entire sample was 41.1 ± 9.7 years.	Both groups followed a regular rehabilitation program (5 times a week), based on endurance exercises, muscle strengthening, stretching, and brain stimulation (twice a week). EXP group, in addition, performed an adapted exercise program twice a week for 90 min, consisting of 30–40 min of Nordic Walking and 50–60 min of strength and balance exercises using same equipment. Interventions were delivered 5 times/week (basic protocol) for 8 weeks, and twice/week for 8 weeks (EXP only).	All patients showed a significant improvement in balance, flexibility, attention and upper and lower limb strength. Differences between EXP and CONT groups did not reach statistical significance.
Straudi et al. 2017 [19]	RCT	Evaluating the effects of VGT vs. BPT on attention, balance and mobility in patients with chronic TBI.	CB&M; UBS; TUG test; GO/Nogo task for attentive functions. Outcomes were recorded pre/post treatment for all patients.	Outpatient setting (Italy).	EXP: 11 patients with chronic TBI and balance deficit (evaluated through CB&M). CONT: 10 patients with chronic TBI and balance deficit (evaluated through CB&M). Mean age of the sample 36 years (12 IQR); mean time since diagnosis 4 years	EXP group received VGT through X-box 360 Kinect platform, while CONT group received a balance platform training. Both interventions were delivered 3 times/week for 6 weeks.	Both groups significantly improved mobility and balance test at CB&M, while greater significant effects at TUG (p<0.01) and UBS (p<0.01) test were observed in EXP group. Static balance test revealed no significant difference for oscillation (path length/sway speed), but EXP group had a positive trend when performing tests with open eyes. Selective attention improved significantly in EXP group.
Tefertiller et al. 2019 [20]	RCT	Evaluating the efficacy of a home-based physiotherapy VR intervention on chronic TBI patients’ balance.	CB&M; BESTest; ABC test; PART-O test.	Home setting (USA).	EXP: 31 patients with chronic TBI (at least 1 year by diagnosis) between 18 and 65 years. CONT: 32 patients with chronic TBI (at least 1 year by diagnosis) between 18 and 65 years. Mean age was 48 ± 12.4 years, mean time from injury was 8.3 ± 9.2 years.	EXP group followed a VR training program, while CONT one followed a traditional HEP program. Both training difficulties were set according to CB&M and BESTest scores, dividing patients in basic, intermediate and higher levels. Each training session lasted 30 min,;intervention was carried out for 3–4 times a week for 12 weeks. Follow-up at 24 weeks.	No significant inter-group difference emerged for CB&M and BESTest scores, with a positive trend for both EXP and CONT. Similarly, ABC and PART-O tests did not reveal any significant between-group difference. All improvements in both groups were maintained at follow-up.
Williams et al. 2022 [21]	Multicenter RCT	Evaluating the efficacy of a 3-month ballistic resistance training, targeted at three lower limbs’ muscle groups, confronting with a not-ballistic exercise program. The study aimed to highlight the best approach in terms of mobility, balance and strength.	HiMAT tool; walking speed at 10-m walking test; Single Leg Stance test; AQoL-6D.	Outpatient setting (New Zealand, Australia).	EXP: 70 ambulatory subjects with subacute TBI (less than a year since diagnosis) with mobility limitation at HiMAT scale, aged 15–65 years. CONT: 74 ambulatory subjects with subacute TBI (less than a year since diagnosis) with mobility limitation at HiMAT scale, aged 15–65 years.	EXP group received a ballistic resistance training conformed to ACSM guidelines; targets for strength exercise were ankle plantar flexors, hip flexors and hip extensors, while knee extensors were targeted for power absorption. Program was tailored based on the initial strength levels detected. CONT group received usual balance training, gait rehabilitation, lower limb stretching/strengthening, and cardiovascular activity. Each session lasted 60 min; intervention was carried out 3 times/week for 12 weeks. Follow up 3 months after training completion.	After the protocol and at 3 months, EXP group had a 3-point favorable difference at HiMAT score. Ballistic training reached a further positive effect when the patient started with a baseline HiMAT score lower than 27. All secondary outcomes showed similar effect in both groups.
Sheridan et al. 2021 [22]	Case Report	Describe feasibility of gait training with RAS in adult patients with TBI.	Adherence; BORG RPE; CB&M; 6MWT; and spatiotemporal params of gait assessed through a pressure-sensitive mat in three conditions (preferred paced, maximum paced, dual task); satisfaction questionnaire.	Community setting (Canada).	Two chronic TBI patients of 42 and 54 years, able to walk autonomously for 10 ms and to provide consent	Training plan provided a RAS protocol developed by a music therapist, while patients trained in the following areas: gait preparation, task-specific training and advanced competencies (gait on different surface, stairs, and stepping over obstacles). Each session lasted 30 min; intervention was carried out 3 times/week for 3 weeks. Follow-up at 1 week (4 by protocol starting).	Training was deemed feasible and with positive effect on gait, balance, speed, coordination, mood by the patients; also, perceived exertion was low. Both patients improved walked distance at 6MWT post-intervention; patient 1 exceeded the MCD. CB&M had a 6-point variation, which did not exceed MCD, in patient 1, and a 1-point variation in patient 2.

## Data Availability

All relevant data are present within the manuscript.

## Supplementary Materials

The following supporting information can be downloaded at: https://www.mdpi.com/article/10.3390/medicina62081453/s1, Table S1: Search strategy.

## Abbreviations

The following abbreviations are used in this manuscript:

TBI	Traumatic brain injury
VR	Virtual reality
VGT	Video game therapy
RAS	Rhythmic auditory stimulation
