# Supplementary material for: Balance Rehabilitation in Patients with Traumatic Brain Injury: A Scoping Review
Source: Medicina (Kaunas). 2026 Jul 27;62(8):1453. doi: 10.3390/medicina62081453 (PMC13514990; doi:10.3390/medicina62081453)
Supplement: Supplementary file 1 [file medicina-62-01453-s001.zip › medicina-4385008-supplementary.pdf]

Table S1. Search strategy.

**Database**	**Search string**
PubMed	(("Brain Injuries, Traumatic"[Mesh]) OR (Brain Injury, Chronic[MeSH Terms]) OR (Brain Damage, Chronic[MeSH Terms]) OR (brain injur*) OR (head injur*) OR (Craniocerebral Trauma[MeSH Terms]) OR (Head Injuries, Closed[MeSH Terms]) OR (Head Injuries, Penetrating[MeSH Terms]) OR (Brain Injuries[MeSH Terms]) OR (Brain Injury, Chronic[MeSH Terms]) OR (Brain Injuries, Diffuse[MeSH Terms]) OR (head injury) OR (head trauma) OR (TBI) OR (mTBI) OR (Intracranial Injuries)) AND ((postural balance[MeSH Terms]) OR (balance disorder*) OR (postural imbalance) OR (postural stability) OR (Impaired balance) OR (Dynamic Balance) OR (postural control) OR (Proprioception[MeSH Terms])) AND ((Neurological Rehabilitation[MeSH Terms]) OR (Rehabilitation[MeSH Terms]) OR (Exercise Therapy[MeSH Terms]) OR (physical therapy) OR (Physical Therapy Modalities[MeSH Terms]) OR (physiotherapy) OR (muscle strengthening) OR (dynamic trunk control) OR (static trunk control) OR (trunk exercises) OR (proprioceptive tablet) OR (virtual reality) OR (VR) OR (core stability training) OR (gait training) OR (balance exercises) OR (lower limb stretching) OR (Dual-Task) OR (kinesiotherapy techniques) OR (aerobic exercise)) AND ((outpatient) OR (inpatient) OR (hospital) OR (day hospital) OR (day service) OR (Hospitalized) OR (Hospital setting) OR (Ambulatory) OR (Clinic) OR (Ambulatory setting) OR (Rehabilitation center) OR (Domiciliary care) OR (Home rehabilitation) OR (Home care) OR (Rehabilitation setting)) NOT ((Brain Concussion[MeSH Terms]) NOT (Concussion) NOT (Cerebral Palsy))
PEDro	brain injury balance rehabilitation
Scopus	TITLE-ABS-KEY(("brain injur*" OR "head injur*" OR "head trauma" OR "craniocerebral trauma" OR "brain damage" OR "brain injuries" OR "traumatic

	brain injury" OR "TBI" OR "mTBI" OR "intracranial injur*")) AND TITLE-ABS-KEY(("postural balance" OR "balance disorder*" OR "postural control" OR "postural imbalance" OR "postural stability" OR "impaired balance" OR "dynamic balance" OR "proprioception")) AND TITLE-ABS-KEY(("neurological rehabilitation" OR "rehabilitation" OR "exercise therapy" OR "physical therapy" OR "physiotherapy" OR "physical therapy modalities" OR "muscle strengthening" OR "trunk control" OR "trunk exercises" OR "virtual reality" OR "VR" OR "core stability training" OR "gait training" OR "balance exercises" OR "dual-task" OR "kinesiotherapy" OR "aerobic exercise" OR "lower limb stretching" OR "proprioceptive tablet")) AND ALL FIELDS (("outpatient" OR "inpatient" OR "hospital" OR "day hospital" OR "clinic" OR "hospitalized" OR "ambulatory" OR "rehabilitation center" OR "home rehabilitation" OR "home care" OR "domiciliary care" OR "rehabilitation setting")) AND NOT TITLE-ABS-KEY(("brain concussion" OR "concussion" OR "cerebral palsy"))
Embase	(('traumatic brain injury'/exp OR 'chronic brain injury'/exp OR 'chronic brain damage'/exp OR brain) AND injur*:ab,ti OR head) AND injur*:ab,ti OR 'craniocerebral trauma'/exp OR 'closed head injury'/exp OR 'penetrating head injury'/exp OR 'brain injury'/exp OR 'diffuse brain injury'/exp OR 'head injury':ab,ti OR 'head trauma':ab,ti OR tbi:ab,ti OR mtbi:ab,ti OR 'intracranial injury'/exp) AND (('postural balance'/exp OR balance) AND disorder*:ab,ti OR 'postural imbalance':ab,ti OR 'postural stability':ab,ti OR 'impaired balance':ab,ti OR 'dynamic balance':ab,ti OR 'postural control':ab,ti OR 'proprioception'/exp) AND ('neurological rehabilitation'/exp OR 'rehabilitation'/exp OR 'exercise therapy'/exp OR 'physical therapy'/exp OR 'physical therapy modalities'/exp OR physiotherapy:ab,ti OR 'muscle strengthening':ab,ti OR 'dynamic trunk control':ab,ti OR 'static trunk control':ab,ti OR 'trunk exercises':ab,ti OR 'proprioceptive tablet':ab,ti OR 'virtual reality'/exp OR vr:ab,ti OR 'core stability training':ab,ti OR 'gait training':ab,ti OR 'balance exercises':ab,ti OR 'lower limb stretching':ab,ti OR 'dual task':ab,ti OR 'kinesiotherapy':ab,ti OR 'aerobic exercise':ab,ti) AND (outpatient:ab,ti OR inpatient:ab,ti OR hospital:ab,ti OR 'day hospital':ab,ti OR 'day service':ab,ti OR hospitalized:ab,ti OR 'hospital setting':ab,ti OR ambulatory:ab,ti OR

	clinic:ab,ti OR 'ambulatory setting':ab,ti OR 'rehabilitation center':ab,ti OR 'domiciliary care':ab,ti OR 'home rehabilitation':ab,ti OR 'home care':ab,ti OR 'rehabilitation setting':ab,ti) NOT 'brain concussion'/exp NOT 'cerebral palsy'/exp
Web of Science	TOPIC=(brain injur* OR head injur* OR brain damage OR head trauma OR craniocerebral trauma OR closed head injur* OR penetrating head injur* OR diffuse brain injur* OR traumatic brain injur* OR TBI OR mTBI OR intracranial injur*) AND TOPIC=(postural balance OR balance disorder* OR postural imbalance OR postural stability OR impaired balance OR dynamic balance OR postural control OR proprioception) AND TOPIC=(neurological rehabilitation OR rehabilitation OR exercise therapy OR physical therapy OR physiotherapy OR muscle strengthening OR dynamic trunk control OR static trunk control OR trunk exercises OR proprioceptive tablet OR virtual reality OR VR OR core stability training OR gait training OR balance exercises OR lower limb stretching OR dual-task OR kinesiotherapy OR aerobic exercise) AND TOPIC=(outpatient OR inpatient OR hospital OR day hospital OR day service OR hospitalized OR hospital setting OR ambulatory OR clinic OR ambulatory setting OR rehabilitation center OR domiciliary care OR home rehabilitation OR home care OR rehabilitation setting) NOT TS=("brain concussion") NOT TS=("cerebral palsy")
